# Thermal Behaviour of Common Thermoresponsive Polymers in Phosphate Buffer and in Its Salt Solutions

**DOI:** 10.3390/polym13010090

**Published:** 2020-12-28

**Authors:** Łukasz Otulakowski, Maciej Kasprów, Aleksandra Strzelecka, Andrzej Dworak, Barbara Trzebicka

**Affiliations:** Centre of Polymer and Carbon Materials, Polish Academy of Sciences, M. Curie-Skłodowskiej 34, 41-819 Zabrze, Poland; lotulakowski@cmpw-pan.edu.pl (Ł.O.); mkasprow@cmpw-pan.edu.pl (M.K.); astrzelecka@cmpw-pan.edu.pl (A.S.); adworak@cmpw-pan.edu.pl (A.D.)

**Keywords:** thermoresponsive polymers, poly(*N*-isopropylacrylamide), poly[oligo(ethylene glycol) methacrylate], polyoxazoline, phosphate buffer, salts, aggregation, solution chemistry

## Abstract

Thermoresponsive polymers are a promising material for drug nanocarrier preparation, which makes the study of their aggregation in physiological conditions very important. In this paper, the thermal behaviour of the thermoresponsive polymers poly(*N*-isopropylacrylamide), poly(2-isopropyl-2-oxazoline-*co*-2-n-propyl-2-oxazoline) and poly[(2-hydroxyethyl methacrylate)-*co*-oligo(ethylene glycol) methyl ether methacrylate] were studied in phosphate buffer (PBS) and solutions of its salts in concentration as in PBS. The thermal response of the polymers was measured using UV-Vis and dynamic light scattering (DLS). The salts shifted the cloud point temperature (T_CP_) of the (co)polymers to higher values compared to the T_CP_ of aqueous polymer solutions. In PBS and NaCl solutions, all polymers exhibited an unexpected and previously unreported transmittance profile. During heating, an additional aggregation of polymers appeared above the T_CP_ accompanied by the formation of a precipitate. In monosodium phosphate solutions and pure water, the studied polymers showed lower critical solution temperature (LCST-type) behaviour. DLS measurements showed that a salt influenced the size of the resulting polymer particles. The sizes and stability of particles depended on the heating rate. In PBS and NaCl solutions, the size of particles in the dispersion decreased above 60 °C, and the precipitate appeared on the bottom of the cuvette. The additional aggregation of polymer and its falling out of solution may hinder the removal of carriers from the body and has to be taken into account when preparing nanocarriers.

## 1. Introduction

Thermoresponsive polymers have been studied for several decades because of their unique properties of undergoing reversible soluble–insoluble or insoluble–soluble phase transitions in solution in response to changes in environmental temperatures. A transition appears above a certain temperature called the cloud point temperature (T_CP_). Lower critical solution temperature (LCST-type) thermoresponsive polymers, in a dilute aqueous solution above T_CP_, form well-defined spherical particles called mesoglobules [1,2,3]. The size of the mesoglobules depends on various factors such as the polymer molar mass, solution concentration, heating protocol and the presence of additives such as salts [4] and surfactants [5,6,7]. Of the group of polymers that undergo a thermal transition during heating, the most thoroughly examined are poly(*N*-isopropylacrylamide) (PNIPAM) [8,9,10,11], polyoxazolines [12,13,14,15] and oligo(ethylene glycol) methacrylates (OEGMA) (co)polymers [16,17,18].

It is well known that the salts present in a thermoresponsive polymer solution may alter its T_CP_ [19,20,21]. Extensive studies on the influence of both organic and inorganic salts on the behaviour of macromolecules in a salt solution were done by Hofmeister [22]. All salts have been divided into one of two groups: kosmotropic and chaotropic salts, which cause “salting in” and “salting out” effects, respectively. In the case of a polymer solution, the presence of salts changes the hydration sphere of polymer chains and, in consequence, changes the polymer solubility. Traditionally, the relative effects of anions present in solution on the physical behaviour of polymers have been ranked according to the Hofmeister series: CO_3_^2−^  >  SO_4_^2−^  >  S_2_O_3_^2−^  >  HPO_4_^2−^  >  H_2_PO_4_^−^  >  F^−^  >  Cl^− ^  >  Br^−^ >  NO_3_^−^  >  I^−^  >  ClO_4_^−^ >  SCN^−^. The order of the Hofmeister series is valid only for a uniform concentration of anions. Chaotropic anions, such as SCN^−^, ClO_4_^−^ and I^−^, show a “salting in” effect leading to an increase of T_CP_; i.e., an increase of polymer solubility. Chaotropic salts in a water solution can disrupt the hydrogen bonding network between water molecules. This affects the stability of the state of macromolecules in solution by weakening the hydrophobic effect. Consequently, water molecules favorably interact with the polymers or proteins and stabilise the intramolecular interactions within these macromolecules [23]. On the other hand, kosmotropic anions lead to a “salting out” effect that arises from a decrease of the T_CP_ of the thermoresponsive polymers (a decrease of polymer solubility). Kosmotropic salts contribute to the stability and structure of water–water interactions. Both “salting in” and “salting out” effects are enhanced by an increase in salt concentration [24].

The influence of the addition of salt on the shifting transition temperature of the thermoresponsive polymers has been previously studied for PNIPAM [25], poly(2-ethyl-2-oxazoline) [26], poly(2-n-propyl-2-oxazoline) [26], poly(2-isopropyl-2-oxazoline) [26], poly[oligo(ethylene glycol) methacrylate] [20] and others [27]. There are only rare cases in which the behaviour of thermoresponsive polymers in the presence of salts during heating above T_CP_ has been reported [28]. Although changes of the thermoresponsive polymers’ T_CP_ values in the presence of salts have been investigated [4,19,25,26], there are no studies on the effect of salts on the aggregation process and particle formation. Recently, Gubarev et al. [29] demonstrated the rigidity changes of poly(2-ethyl-2-oxazoline)s chains during heating in physiological conditions.

Bruce and coworkers [30] used molecular dynamic simulations to explain the complex behaviour of PNIPAM in a solution of a mixture of two salts, namely NaCl and NaSO_4_. The collapse and subsequent swelling processes of PNIPAM in solution with increasing salt concentrations were ascribed to changes in the water affinity of anions present in the solution and an increase in the ion-pairing affinity between SO_4_^−^ and Na^+^. The authors showed that the effect of salts on the T_CP_ of PNIPAM was non-additive.

The high potential of thermoresponsive polymers as drug carriers results from the possibility of controlling the thermal aggregation leading to the formation of particles; a second important aspect is the simple procedure of the stabilisation of the particles obtained as a result [31,32,33,34]. In this regard, the behaviour of thermoresponsive polymers in solutions containing components present in the biological medium is essential. The medium usually used in biological studies is phosphate buffer (PBS). PBS is isotonic and non-toxic to most cells and is used as a medium to simulate physiological pH in different research studies. However, the behaviour of thermoresponsive polymers in PBS is seldom described in the literature [4,19,25,35,36].

Several studies have found that the behaviour of thermoresponsive polymers in PBS solution is typical for LCST-type polymers [35,36]. Nishimori et al. [35] reported transmittance changes of PNIPAM in a 5 mM PBS solution. The temperature-dependence curve of transmittance was sigmoidal. Nagase et al. studied the thermoresponsive behaviour of NIPAM and *N,N*′-dimethylaminopropyl acrylamide or *N,N*′-dimethylacrylamide copolymers in Dulbecco’s phosphate saline, which has a more complex composition than traditional PBS [36]. The polymer transmittance plots did not indicate changes in transmittance above T_CP_, and the curve was also sigmoidal.

However, other studies have shown that a subsequent aggregation and precipitation of polymers above T_CP_ were observed [37,38]. Longenecker et al. [37] described the thermoresponsive properties of various copolymers of HEMA and cationic monomers. In the case of poly[(2-hydroxyethyl) methacrylate-*co*-methacrylic acid] in aqueous solution containing sodium chloride, the authors observed an increase of solution transmittance during heating above the T_CP_ and the appearance of a sludge. A similar behaviour of poly[(ethylene glycol) methyl ether acrylate-*co*-(methoxy ethoxy ethyl) acrylate] in PBS solution was reported but not discussed by the authors [38].

In our previous work [39], the synthesis and thermal behaviour of a set of poly[(2-hydroxyetyl)methacrylate-*co*-oligo(ethylene glycol) methacrylate] (P(HEMA-*co*-OEGMA_300_)) copolymers weredescribed. In PBS, the copolymers showed thermal transitions followed by a significant transmittance increase above T_CP_. The increase of transmittance was accompanied by additional polymer aggregation and the formation of a transparent gel.

In most cases, the envisaged application of thermoresponsive polymers is in medicine and biology, where the polymer particles work in physiological solutions of a complex composition.

Possible future applications in medicine, our previous results and the gap in the reported literature motivated us to systematically study the temperature behaviour and aggregation of representative thermoresponsive (co)polymers poly(*N*-isopropylacrylamide) (PNIPAM), poly(2-isopropyl-2-oxazoline-*co*-2-n-propyl-2-oxazoline) (P(IPO-*co*-NPO)) and poly[2-hydroxyethyl methacrylate-*co*-oligo(ethylene glycol) methyl ether methacrylate] (P(HEMA-*co*-OEGMA_300_)) in phosphate buffer solutions. We have also tested the aforementioned polymers in solutions of a single salt presented in PBS to determine which salts are responsible for the formation of the observed polymer precipitation upon heating above T_CP_. The aggregation process of thermoresponsive chains and the sizes of particles formed under slow and abrupt heating were discussed.

## 2. Materials and Methods

### 2.1. Materials

Phosphate buffer saline 10× concentrate (PBS) (after dilution c = 0.1 M, containing sodium phosphate and sodium chloride as supplier inform), monosodium phosphate monohydrate ≥99.0% (NaH_2_PO_4_ * H_2_O), disodium phosphate heptahydrate 98.0–102.0% (Na_2_HPO_4_ * 7H_2_O), and sodium chloride ≥99.5% (NaCl) were purchased from Sigma-Aldrich (Hamburg, Germany) and used as received. PNIPAM (M_n_ = 67,000 g/mol and M_w_/M_n_ = 1.8) was purchased from Sigma-Aldrich (Hamburg, Germany) and used as received. P(IPO-*co*-NPO) (M_n_ = 51,000 g/mol and M_w_/M_n_ = 1.3) was synthesised via cationic ring-opening polymerisation initiated by methyl 4-nitrobenzenesulfonate at the Centre of Polymer and Carbon Materials, Polish Academy of Sciences, as previously shown in [40]. P(HEMA-*co*-OEGMA_300_) with 79.7% mol of HEMA and 20.3% mol of OEGMA_300_ and M_n_ = 33,000 g/mol (M_w_/M_n_ = 1.2) was synthesised at the Centre of Polymer and Carbon Materials at the Polish Academy of Sciences via atom transfer radical polymerisation as described in [39]. The molar mass of polymers was determined using gel permeation chromatography with a multiangle light scattering detector, which was considered to deliver their absolute values.

### 2.2. Methods

#### 2.2.1. Phosphate Buffer and Salt Solutions Preparation

The water used to obtain solutions was purified using a commercial ion exchange system (Hydrolab, Straszyn, Poland).

Individual salt solution concentrations corresponding to their concentration in commercial PBS were used. In total, 0.737 g (6.14 mmol) of monosodium phosphate was placed in a 250 mL volumetric flask and dissolved in 200 mL of deionised water. The solution was placed on a mechanical shaker overnight to ensure that the salt completely dissolved into the solution. The next day, water was added to achieve a final volume of 250 mL. The final concentration of NaH_2_PO_4_ in solution was 0.02456 M.

The solutions of disodium phosphate and sodium chloride were prepared in the same manner using 2.676 g Na_2_HPO_4_ (18.85 mmol) and 2.018 g NaCl (34.5 mmol). The final concentrations were 0.0754 M for the Na_2_HPO_4_ solution and 0.137 M for the NaCl solution.

#### 2.2.2. Polymer Solutions Preparation

Each studied polymer was dissolved in water, PBS and the single salt solutions. In all solutions, the polymer concentration was 0.5 g/L. To ensure complete dissolution of the polymers, the solutions were placed in a refrigerator at 4 °C overnight.

#### 2.2.3. Turbidity Measurements

The T_CP_ values in the prepared solutions were determined using a SPECORD 200 PLUS UV-Vis spectrophotometer (Analytik Jena) (Jena, Germany) with a Peltier temperature-controlled cell holder. The samples were measured at a constant transmittance wavelength (λ = 550 nm). The polymer solutions at a concentration of 0.5 g/L were heated from 20 °C to 70 °C at a heating rate of 1 °C/min. Afterwards, the samples were cooled to 25 °C at a cooling rate of 1 °C/min. The T_CP_ value was determined by the temperature at which the transmittance of the polymer solution reached 50% of the difference of transmittance above and below the transition.

#### 2.2.4. Characterisation by Dynamic Light Scattering

Dynamic light scattering was used to follow the aggregation process that occurred under the influence of temperature and to determine the sizes of the aggregated structures. The measurements were recorded using a Brookhaven BI-200 (Brookhaven Instruments Corporation, Holtsville, NY, USA) goniometer with vertically polarised incident light of wavelength *λ* = 637 nm (semiconductor laser diode 36 mW) and equipped with a Brookhaven BI-9000 AT digital autocorrelator (Brookhaven Instruments Corporation, Holtsville, NY, USA). The intensity of scattered light was measured at an angle of 90°. The autocorrelation functions were analysed using the constrained regularised inverse Laplace transform (CONTIN program) method to obtain the distributions of the relaxation rates (*Γ*). The latter provided distributions of the apparent diffusion coefficient *D* (*D* = *Γ*/*q*^2^, where *q* is the magnitude of the scattering vector, *q* = (4*πn*/*λ*) sin (*θ*/2), and *n* is the refractive index of the medium). The apparent hydrodynamic radius ($R_{h}^{90}$) was obtained from the Stokes–Einstein equation (Equation (1)):

(1) $$R_{h}^{90}=\frac{kT}{6\pi \eta D}$$

for *θ* = 90°, where *k* is the Boltzmann constant and *η* is the viscosity of water at temperature *T*. The dispersity (D) of the particle diameters was given as $\mu_{2}/{\bar{\Gamma }}^{2}$, where $\bar{\Gamma }$ is the average relaxation rate and *µ*_2_ is its second moment.

The rate of heating used in slow heating DLS experiments ranged between 0.3 and 0.5 °C/min.

## 3. Results and Discussion

Because of their easily tailored properties, response to thermal stimuli in the normal human body temperature range and biocompatibility, PNIPAM, polyoxazolines and HEMA copolymers were considered to be of interest as potential drug carriers.

*N*-isopropylacrylamide was used to synthesise many thermoresponsive (co)polymers. Some of these copolymers were conjugated with bioactive species, such as peptides, proteins and drugs [41,42,43,44,45]. The thermal behaviours of PNIPAM and its copolymers have been widely studied in water but rarely tested in PBS [44,46,47].

The thermal and crystalline properties of thermoresponsive poly(2-oxazoline)s have been the subject of many works [48,49,50]. The random P(IPO-*co*-NPO) has been examined in a water environment and exhibited no hysteresis or second aggregation upon heating above T_CP_ [51].

Thermoresponsive P(HEMA-*co*-OEGMA) has been synthesised by our group and characterised with special attention paid to its behaviour in water and PBS solutions. The control of the HEMA content in the copolymer allowed us to tune the T_CP_ [39].

As was discussed in the introduction, the properties of thermoresponsive polymers in water solutions have been the subject of numerous studies. However, their behaviour in a salt environment similar to that found in the human body has still not been studied in detail and is not fully understood.

### 3.1. Turbidity Studies

The thermal behaviours of thermoresponsive PNIPAM, P(IPO-*co*-NPO) and P(HEMA-*co*-OEGMA_300_) with 79.7%mol of HEMA and 20.3%mol of OEGMA_300_ were studied using UV-Vis in PBS and its individual salt solutions. The heating from 20 °C to 70 °C (well above the T_CP_ of studied polymers) and then cooling procedures were used. The experiments were repeated 3–5 times with preprepared samples. The transmittance was measured for the solutions with a total polymer concentration of 0.5 g/L. To determine the effect of individual salts on the behaviour of the polymers, solutions with salt concentrations corresponding to the concentration in PBS were used.

In addition, polymers were tested in pure water according to the same procedures as those used for salt solutions to compare the transition temperatures in the respective media. The transmittance vs. temperature curves for the polymers in water were sigmoidal (Figure S1). The cooling curves almost overlapped with the heating curves, indicating full reversibility of thermal aggregation. The T_CP_ values determined for the polymers in water are given in Table 1. As can be expected, the obtained T_CP_ values are in good agreement with those previously reported [39,40,52].

**Figure 1:**
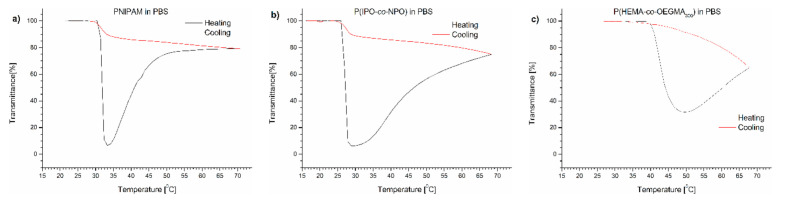
Transmittance versus temperature curves of PBS solutions of (**a**) PNIPAM, (**b**) P(IPO-*co*-NPO) and (**c**) P(HEMA-*co*-OEGMA_300_). Total solute concentration: 0.5 g/L; heating/cooling rate: 1 °C/min. Heating: black; cooling: red.

The course of the transmittance curves shown in Figure 1 indicates the polymers behaved quite differently when placed in PBS.

The transmittance curves of each of the polymers tested were not sigmoidal during heating, but after passing T_CP,_ the curve reached a minimum. A further elevation of temperature led to a pronounced increase of transmittance of the dispersions. The dispersion became transparent, indicating a decrease of the particle number in the volume “visible” by the UV beam. The dispersions were not allowed to anneal at high temperatures but cooled immediately after reaching 70 °C. The transmittance curves upon cooling at the same rate (1 °C/min) did not follow the profile of the heating curves and lay greatly above the latter. The transmittance increased to the initial value with decreasing temperature.

**Figure 2:**
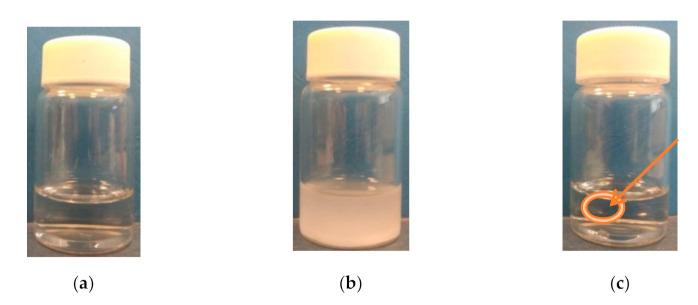
Solution of P(HEMA-*co*-OEGMA_300_) in PBS at different temperatures: (**a**) room temperature, (**b**) cloud point temperature (**c**) at 70 °C (the arrow and circle show the formed gel). Concentration of solution: 0.5 g/L.

It was observed that when the temperature increased above the transition temperature, the (co)polymers started to precipitate (Figure 2 and Figure S2). The precipitate was stable at elevated temperatures and dissolved only after storing the vials in the fridge.

Figure 2 shows the changes in the P(HEMA-*co*-OEGMA_300_) solution in PBS during heating. A transparent solution was observed at room temperature (Figure 2a). Figure 2b depicts the cloudy polymer dispersion achieved just above T_CP_. An increase in the temperature above the minimum of transmittance led to a clearing of the dispersion and formation of a gel (Figure 2c).

The T_CP_ of the (co)polymers in PBS was determined as the value at which the transmittance of the polymer solution reached 50% of the value between the maximum and the minimum of transmittance. The T_CP_ values are presented in Table 1. In all cases, the transition temperatures of the polymers in PBS were shifted toward lower temperatures compared to pure water. The highest shift of 3 °C was observed for PNIPAM. The lowering of the T_CP_ indicates that water–polymer interactions in PBS were replaced to some extent by the salt interactions with water, causing a “salting out” effect.

**Figure 3:**
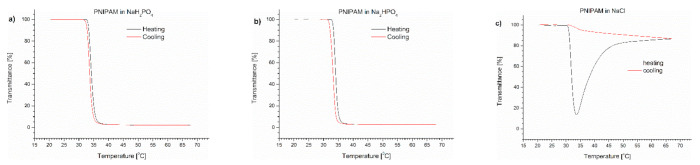
Transmittance versus temperature curves of solutions of PNIPAM in (**a**) monosodium phosphate, (**b**) disodium phosphate, (**c**) NaCl. Total solute concentration: 0.5 g/L; heating/cooling rate: 1 °C/min. Heating: black; cooling: red.

**Figure 4:**
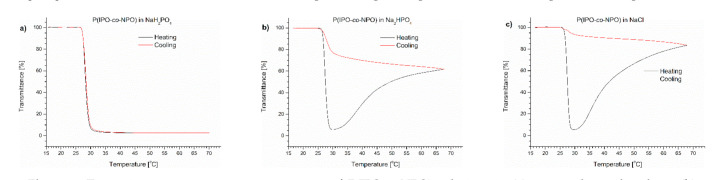
Transmittance versus temperature curves of P(IPO-*co*-NPO) solutions in (**a**) monosodium phosphate, (**b**) disodium phosphate, (**c**) NaCl. Total solute concentration: 0.5 g/L; heating/cooling rate: 1 °C/min. Heating: black; cooling: red.

**Figure 5:**
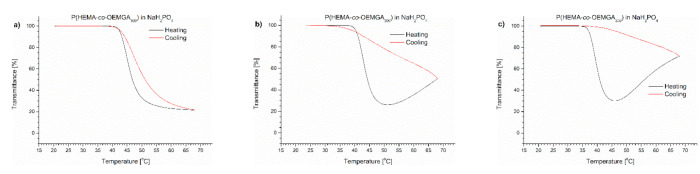
Transmittance versus temperature curves of solutions of P(HEMA-*co*-OEGMA_300_) in (**a**) monosodium phosphate, (**b**) disodium phosphate, (**c**) NaCl. Total solute concentration: 0.5 g/L; heating/cooling rate: 1 °C/min. Heating: black; cooling: red.

To determine which salt was responsible for the effect of polymer precipitation upon heating, we performed a series of experiments to follow the influence of individual Na_2_HPO_4_, NaH_2_PO_4_ and NaCl salts on the (co)polymers’ behaviour. The content of the salts in the studied polymer solutions corresponded to their concentration in PBS. The transmittance of PNIPAM, P(IPO-*co*-NPO) and P(HEMA-*co*-OEGMA_300_) solutions containing salts are shown in Figure 3, Figure 4 and Figure 5, respectively.

In the case of PNIPAM dissolved in salt solutions, a minimum on the transmittance curve and additional aggregation at the temperature above were only observed in the presence of NaCl (Figure 3c). The T_CP_ values in phosphate salts were slightly shifted towards lower temperatures in comparison with T_CP_ in water, but were higher than in PBS (Table 1). The T_CP_ of PNIPAM in NaCl solution was 32 °C, which is equal to the value measured in PBS. An insignificant hysteresis was observed for phosphate PNIPAM solutions, whereas for NaCl, the return curve was similar to that seen in PBS.

The P(IPO-*co*-NPO) copolymer in a monosodium phosphate solution showed a typical LCST-type transition. The transmittance curves for the heating and cooling procedures were sigmoidal and almost overlapped. A precipitation was not observed. The transition temperature of the copolymer in this solution was 0.5 °C lower than in water. The T_CP_ values of the polyoxazoline in Na_2_HPO_4_ and NaCl solutions were lower than that for the NaH_2_PO_4_ solution (Table 1). Bloksma et al. [26] reported the “salting out” effect for a set of oxazoline (co)polymers in NaCl solution. The T_CP_ depended strongly on the salt concentrations. The nature of the influence of salts on the transition temperature was independent of the type of oxazoline. However, the transmittance curves of polyoxazoline in the presence of Na_2_HPO_4_ and NaCl resembled those observed for the polymer solution in PBS. After the decrease of transmittance related to an LCST-type transition, the curves went through the minimum and, with a further increase of temperature, a whitish precipitate was formed at the bottom of the vial (Figure 2). When comparing both salts, the effect of the precipitate formation was stronger in the NaCl solution at 70 °C, and the observed transmittance (84%) exceeded the value of the dispersion of polyoxazoline in the presence of Na_2_HPO_4_.

In the case of P(HEMA-*co*-OEGMA_300_) with 79.7%mol of HEMA and 20.3%mol of OEGMA_300_, the presence of salts in the solution caused a decrease of T_CP_ in the order NaH_2_PO_4_ (0.02456 M) < Na_2_HPO_4_ (0.0754 M) < NaCl (0.137 M) < pure water solution (Table 1). In Na_2_HPO_4_ and NaCl solutions, the copolymer precipitation was observed when the temperature was raised above the minimal value (Figure 5 and Figure S2). In the solution of monosodium phosphate, a pronounced hysteresis was visible during the cooling cycle, indicating a possible occurrence of a minimal particle precipitation.

For the all studied salt solutions, except for NaH_2_PO_4,_ the precipitation of the thermoresponsive polymers was found. The concentration of salts used in the experiments was imposed by their concentration in PBS. It was clearly shown that NaCl had the most significant influence on polymer aggregation, but this salt also had the highest concentration in PBS. The differences in the concentration of the studied salts meant that the observed dependences did not fit the salts’ relations in the Hofmeister series.

The results of the experiments proved that the influence of salts on polymer aggregation was non-additive.

It should be emphasized that polymer structures formed in salt solutions heated above the minimum cannot be regarded as thermodynamically stable. When the dispersions were held at the selected temperature for a longer time, the transmittance slowly increased.

In separate experiments, the PBS solutions of the studied polymers were heated only to temperatures slightly (1–2 °C) below the minimum value observed on the transmittance curves. This was similar to that observed previously for poly[2-hydroxyethyl methacrylate-*co*-oligo(ethylene glycol) methacrylate] by Kasprów et al. [39]. All of the (co)polymers studied using this procedure showed reversible cloudy-to-transparent phase behaviour with no signs of precipitation.

### 3.2. Aggregates Formed by Thermoresponsive (Co)Polymers in PBS and Salt Solutions

The light scattering experiments of (co)polymer solutions with a concentration of 0.5 g/L were performed in PBS and salts present in PBS in the temperature range from 20 to 70 °C. The behaviour of the polymers in water was also studied.

Two protocols—abrupt and slow (gradual) heating—were used. It was noticed that the aggregation of thermoresponsive polymers in the gradual heating process led to particles of sizes significantly larger than those obtained when the solution was heated abruptly. These dependencies were found for a wide range of thermoresponsive polymers, such as copolymers of OEGMA [18] and PNIPAM and its copolymers with a diverse architecture [11,53,54]. An increase of the solution heating rate led to a predominance of intra-chain interactions and caused a decrease in the sizes achieved by the aggregates formed in the solution. Particles were formed from polymer chains presented in a set volume of solution. An increase in the heating rate of the solution reduced the contact time; thus, the likelihood of aggregation also decreased.

#### 3.2.1. Slow Heating Mode

The hydrodynamic diameters (D_h_^90^) of the particles and scattered light intensity (I^90^) were measured at scattering angle of 90°. The results of the DLS studies of the polymer solutions are expressed as D_h_^90^ vs. temperature. On the same plots, changes in scattered light intensity are presented. Slow heating resembled the experimental conditions used in UV-Vis experiments. However, it must be noted that, due to experimental limitations, the rates of heating and cooling in the slow heating protocol differed considerably from those applied in UV-Vis measurements. This caused differences between the experiments, but the results obtained using both methods are complementary.

The diameters of aggregates measured by DLS from all experiments at 70 °C are summarised in Table S1.

In pure water, the sigmoidal D_h_^90^ vs. temperature dependence was observed for all polymers (Figure S3). The particle sizes and the I_90_ values remained stable in temperatures above the phase transition, indicating the formation of thermodynamically stable dispersions’ in water. The smallest particles with a hydrodynamic diameter of 770 nm were found for PNIPAM (Table S1).

**Figure 6:**
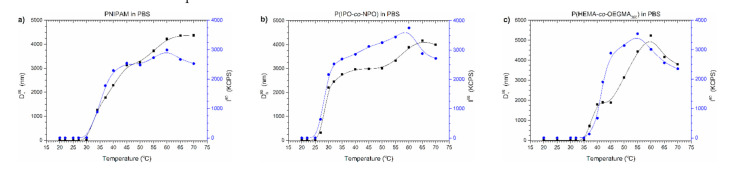
D_h_^90^ and I^90^ versus temperature plots of solution of (**a**) PNIPAM, (**b**) P(IPO-*co*-NPO) and (**c**) P(HEMA-*co*-OEGMA_300_) in commercial PBS. Polymer concentration: 0.5 g/L; gradual heating.

In Figure 6, D_h_^90^ and I_90_ temperature functions of the polymers’ dispersions in PBS are presented.

Two transitions can be easily distinguished on the curves during the heating of thermoresponsive polymers in PBS (Figure 6). In the first phase of the aggregation process, the sizes of particles increased, reflecting LCST-type changes. Then, the sizes of particles stabilised, and after exceeding a certain temperature, the particles began to grow again. This increase in particle sizes was accompanied by a decrease in the intensity of scattered light. The observed dependence indicated that the amount of particles present in dispersion decreased. In the DLS experiment, the formation of sludge at the bottom of the vial was noticed, which caused a decrease in particle concentration and, simultaneously, the lowering of scattered light intensity. The observations agreed with data from UV-Vis studies where the polymer precipitated in PBS above the temperature of the minimum transmittance.

**Figure 7:**
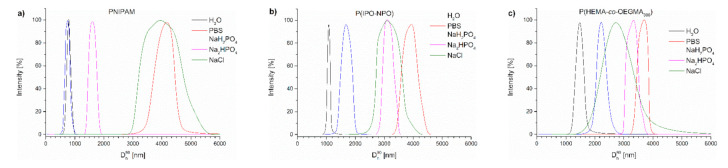
Size distribution of particles of (**a**) PNIPAM, (**b**) P(IPO-*co*-NPO) and (**c**) P(HEMA-*co*-OEGMA) acquired by slow heating in different salt solutions at 70 °C.

Figure 7 contains the distributions of the sizes of aggregates present in the studied polymer dispersions at 70 °C. It can be seen that, in PBS, as a result of heating the polymer chains, particles were formed that were much larger than in water (Table S1, Figure 7).

Further studies were focused on the aggregation of the thermoresponsive polymers in solutions containing only individual salts present in PBS. As in UV-Vis experiments, concentrations of salts were set to match their concentrations in PBS. The results for D_h_^90^ and light intensity as a function of temperature are shown in Figures S4–S6. Diameters of polymer aggregates at 70 °C observed in dispersions after gradual heating are shown in Table S1.

In phosphate salt solution, above the phase transition, PNIPAM formed a stable dispersion (Figure S4a,b). The aggregates did not precipitate when the temperature was increased. In NaH_2_PO_4_, the sizes of aggregates were comparable with the sizes observed in water (Table S1, Figure S3), whereas in Na_2_HPO_4_, the particles were much larger. However, precipitation did not occur. In the NaCl solution, PNIPAM formed large aggregates of 4000 nm immediately after the transition (Figure S4c). This was accompanied by polymer precipitation and the decrease of scattered light intensity. In UV experiments, this behavior was manifested as an increase in transmittance (Figure 2).

The DLS results for P(IPO-*co*-NPO) (Figure S5), as for PNIPAM, were in good agreement with the transmittance observations as detected by UV-Vis. Above the temperature transition, polyoxazoline dispersion was thermodynamically stable in the NaH_2_PO_4_ solution. In NaCl and Na_2_HPO_4_, DLS indicated the progressing precipitation of polyoxazoline accompanied by a lowering of I^90^.

Furthermore, P(HEMA-*co*-OEGMA_300_) particles in a monosodium phosphate exhibited dispersion with heating above the temperature transition (Figure S6a). The dispersions of the copolymer in NaCl and Na_2_HPO_4_ were not thermodynamically stable (Figure S6b,c). The size decrease and the precipitate appearance were observed for both salts and were accompanied by a lowering of the intensity of scattering of light.

All studied polymers in the monosodium phosphate salt formed stable particles which did not precipitate. The same was confirmed for the transmittance experiments (Figure 3, Figure 4 and Figure 5). This could be due to the low concentration of this salt in solution. All copolymer dispersions in NaCl and P(IPO-*co*-NPO) and P(HEMA-*co*-OEGMA dispersions in disodium phosphate) were not stable; their heating above temperature transition led to progressing precipitation.

The changes of particle sizes during heating/cooling runs of thermoresponsive polymer in salt solutions are shown for PNIPAM (Figure S7a–d) as an example. The D_h_ vs. temperature curves reflects a similar dependence to that in UV-Vis spectrophotometric measurements.

#### 3.2.2. Abrupt Heating Mode

**Figure 8:**
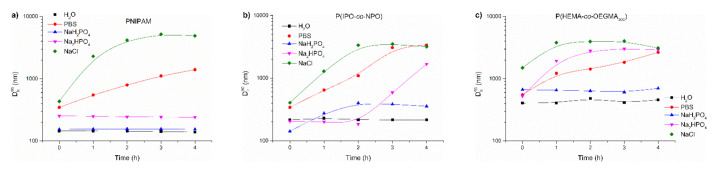
Variations of D_h_^90^ with time for abruptly heated solutions of (**a**) PNIPAM, (**b**) P(IPO-*co*-NPO) and (**c**) P(HEMA-*co*-OEGMA_300_). Polymer concentration: 0.5 g/L; abrupt heating at 70 °C.

It has repeatedly been shown that the abrupt, shock heating of a solution of a thermoresponsive polymer results in the formation of well-defined, relatively small and narrowly distributed mesoglobules in comparison to slow heating [18,55,56]. Salt solutions of the polymers were subjected to abrupt heating from room temperature to 70 °C, which was well above their transition temperature. The size of particles created using this procedure is shown as a function of time in Figure 8 and listed in Table 2. As an example, the particle size distribution of PNIPAM in salt solutions and water at 70 °C is presented in Figure S8a. Figure S8b shows the changes in the particle distribution in NaCl with time.

These measurements evidence the thermodynamic instability of the polymer particles in PBS and indicate which PBS salt/salts are responsible for this behavior.

For PNIPAM, the D_h_^90^ vs. time is shown in Figure 8a. The particles formed by PNIPAM chains in water and phosphate salts were stable over time. On the other hand, PNIPAM particles in commercial PBS and NaCl grew over time, reaching 1400 nm and 4900 nm after 4 h, respectively. However, in sodium chloride solutions, the sizes of aggregates decreased when the time of heating exceeded three hours. This was accompanied by polymer precipitation.

The time behaviour of particles formed by P(IPO-*co*-NPO) in the abrupt heating mode are shown in Figure 8b. In water, P(IPO-*co*-NPO) aggregates of 220 nm were created. For the polymer dissolved initially in PBS, NaCl and Na_2_HPO_4_, the sizes of aggregates increased with time. Particles in PBS reached ca. 3400 nm diameter after 4 h. The polymer formed a precipitate at the bottom of the vial. In a monosodium phosphate solution, aggregates of the polyoxazolines increased to 360 nm and remained stable over time.

As a result of abrupt heating, the chains of P(HEMA-*co*-OEGMA_300_) formed particles of about 3000 nm after 4 h when the copolymer was dissolved in PBS, NaCl and Na_2_HPO_4_ (Figure 8c). In these solutions, a part of the polymer precipitated after 3 h of annealing. The aggregates in water and NaH_2_PO_4_ were stable over time but of a significantly larger size than for other thermoresponsive polymers studied here.

When comparing the influence of individual salts on particles formed in the abrupt mode, it can be noticed that NaH_2_PO_4_ at the concentration used in the experiments did not destabilise the dispersions of polymer mesoglobules. In all cases, they were significantly smaller than in gradual heating, but larger than in water. The particles of the studied polymers were not stable in a 0.134 M NaCl solution and PBS. In these media, the particles grew over time, which led to their precipitation, as in gradual heating.

## 4. Conclusions

In the majority of the reported scientific research, the thermal behaviour of thermoresponsive polymers has been studied in water. The purpose of our research was to follow the temperature response of three chosen representative thermoresponsive polymers—poly(*N*-isopropylacrylamide), poly(2-isopropyl-2-oxazoline-*co*-2-n-propyl-2-oxazoline) and poly[2-hydroxyethyl methacrylate-*co*-oligo(ethylene glycol) methyl ether methacrylate]—in PBS, the medium usually used to simulate physiological pH and used to test polymer behavior in an environment matching that of the human body. Turbidity studies in PBS solutions have shown that the presence of PBS salts lowers the transition temperatures by a few degrees at a polymer concentration of 0.5 g/L. We have observed, however, that the behavior of tested polymers in PBS is different from that in water. The heating of solutions above the temperature of the minimum transmittance led to subsequent polymer precipitation. The precipitate remains stable during cooling and does not dissolve. Irreversible precipitation reduces the concentration of the polymer in the dispersion in the path of light, which increases the transmittance value above the T_CP_. The plots of the heating and cooling cycles do not overlap. To determine which salt was responsible for the precipitation, studies were performed with individual salt solutions at concentrations corresponding to their concentration in commercial PBS. For all studied polymers, the precipitation was observed for a 0.137 M NaCl solution, which was the highest concentration of the tested individual salts. Na_2_HPO_4_ caused the same effect in solutions of polyoxazoline and polymethacrylate, but not of PNIPAM. The resulting precipitates were very durable, but the precipitate dissolved after prolonged storage in temperatures below T_CP_.

The polymers’ aggregate formation followed by DLS allowed the study of the process of the self-assembly of thermoresponsive chains and the determination of the sizes of particles in dispersions during their abrupt and gradual heating. The particle changes during gradual heating correlated well with the process of transmittance changes. Studies showed that the influence of salt on aggregation depends on the salt ion type, its concentration and the polymers’ main chain structure. The results indicated that the effect of the presence of salt is non-additive.

Further detailed research is needed to determine the effect of salt concentration and to explain the phenomenon of the formation of precipitates above the T_CP_ of thermoresponsive polymers in the salt solutions. This is of special importance as thermoresponsive polymers are frequently proposed as drug carriers. The results presented in the paper clearly indicate that, for this purpose, the study of the thermal behavior of thermoresponsive polymers should be carried out in an environment close to the physiological environment.

## Figures and Tables

**Table 1:** Cloud point temperature (T_CP_) of polymers in different solutions depicted from UV-vis traces. PNIPAM: poly(*N*-isopropylacrylamide); P(IPO-*co*-NPO): poly(2-isopropyl-2-oxazoline-*co*-2-n-propyl-2-oxazoline); P(HEMA-*co*-OEGMA_300_): poly[2-hydroxyethyl methacrylate-*co*-oligo(ethylene glycol) methyl ether methacrylate].

	T_CP_ (°C) by UV-Vis				
(Co)Polymer	Water	PBS	NaH_2_PO_4_ Solution	Na_2_HPO_4_ Solution	NaCl Solution
PNIPAM	35.0	32.0	34.0	34.0	32.0
P(IPO-*co*-NPO)	28.5	27.0	28.0	27.5	27.5
P(HEMA-*co*-OEGMA300)	46.0	44.5	45.5	43.0	39.5

**Table 2:** Polymeric particles sizes in different solutions after abrupt heating and 4 h of sample annealing at 70 °C.

	Hydrodynamic Diameter after 4 h at 70 °C (nm)				
Copolymer	Water	PBS	NaH_2_PO_4_ Solution	Na_2_HPO_4_ Solution	NaCl Solution
PNIPAM	140	1400	155	240	4875
P(IPO-*co*-NPO)	220	3360	360	1680	3450
P(HEMA-*co*-OEGMA300)	460	2660	700	2900	3100

## Data Availability

The data presented in this study are available on request from the corresponding author. The data are not publicly available due to them being part of ongoing project.

## Supplementary Materials

The following are available online at https://www.mdpi.com/2073-4360/13/1/90/s1, Figure S1. Transmittance versus temperature curves of aqueous solutions of (a)PNIPAM, (b) P(IPO-co-NPO) (c) P(HEMA-co-OEGMA_300_). Total solute concentration 0.5 g/L, heating/cooling rate 1 °C/min. Heating-black, cooling-red; Figure S2. Representative pictures of precipitates formed in NaCl solution of (a) PNIPAM, (b) P(IPO-co-NPO) (c) P(HEMA-co-OEGMA_300_); Figure SI3. D_h_^90^ versus temperature plots of water solution of (a)PNIPAM, (b) P(IPO-co-NPO), (c) P(HEMA-co-OEGMA_300_). Total solute concentration 0.5 g/L. Heating-black, cooling-red; Table S1. Diameters of polymers aggregates after gradual heating depicted from DLS at 70 °C; Figure S4. D_h_^90^ and I^90^ versus temperature plots of PNIPAM in (a) monosodium phosphate, (b) disodium phosphate, (c) sodium chloride solutions. Polymer concentration 0.5 g/L, gradual heating; Figure S5. D_h_^90^ and I^90^ versus plots of P(IPO-co-NPO) in (a) monosodium phosphate, (b) disodium phosphate, (c) sodium chloride solutions. Polymer concentration 0.5 g/L, gradual heating; Figure S6. D_h_^90^ and I^90^ versus temperature plots of P(HEMA-co-OEGMA_300_) in (a) monosodium phosphate, (b) disodium phosphate, (c) sodium chloride solutions. Polymer concentration 0.5 g/L, gradual heating; Figure S7. D_h_^90^ versus temperature plots during heating and cooling of PNIPAM in (a) commercial PBS (b) disodium phosphate, (c) sodium phosphate (d) sodium chloride solutions. Polymer concentration 0.5 g/L, gradual heating; Figure S8. Particles size distribution of PNIPAM in abrupt heating (a) in different solutions after 4h, (b) in solution of NaCl after different times periods. Table S1. Diameters of polymer aggregates after gradual heating depicted from DLS at 70 °C.
