# Supplementary material for: Thermal Behaviour of Common Thermoresponsive Polymers in Phosphate Buffer and in Its Salt Solutions
Source: Polymers (Basel). 2020 Dec 28;13(1):90. doi: 10.3390/polym13010090 (PMC7795651; doi:10.3390/polym13010090)
Supplement: Supplementary file 1 [file polymers-13-00090-s001.pdf]

Supplementary information

Thermal behaviour of common thermoresponsive polymers in phosphate buffer and in its salt solutions

Łukasz Otulakowski[1], Maciej Kasprów[1], Aleksandra Strzelecka[1], Andrzej Dworak[1], Barbara Trzebicka[1]*

[1]Centre of Polymer and Carbon Materials, Polish Academy of Sciences, M. Curie-Skłodowskiej 34, Zabrze, 41-819, Poland

*Corresponding author: Barbara Trzebicka
e-mail: btrzebicka@cmpw-pan.edu.pl

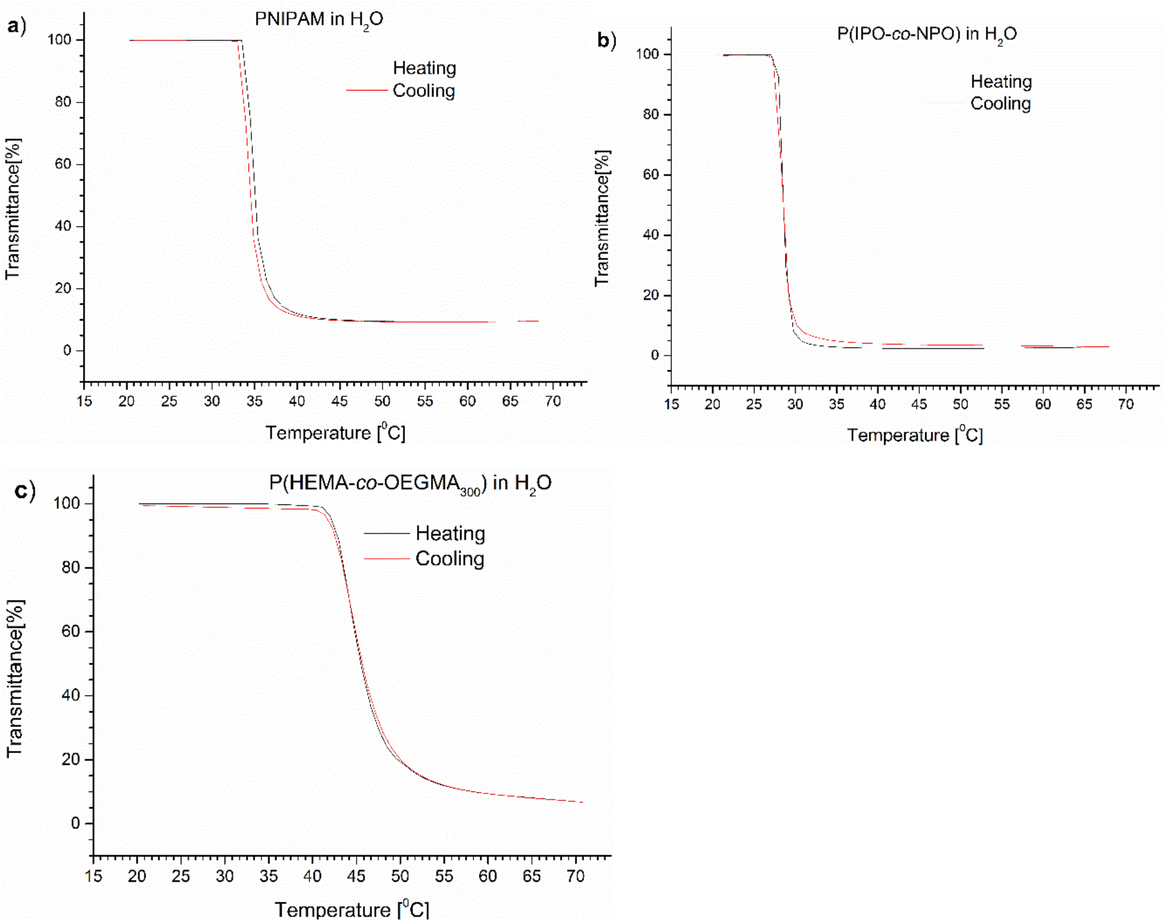

Figure S1. Transmittance versus temperature curves of aqueous solutions of a)PNIPAM, b) P(IPO-*co*-NPO) c) P(HEMA-*co*-OEGMA$_{300}$). Total solute concentration 0.5 g/L, heating/cooling rate 1 °C/min. Heating - black, cooling - red.

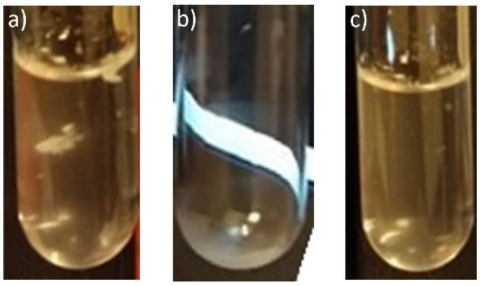

Figure S2. Representative pictures of precipitates formed in NaCl solution of a) PNIPAM, b) P(IPO-*co*-NPO) c) P(HEMA-*co*-OEGMA$_{300}$)

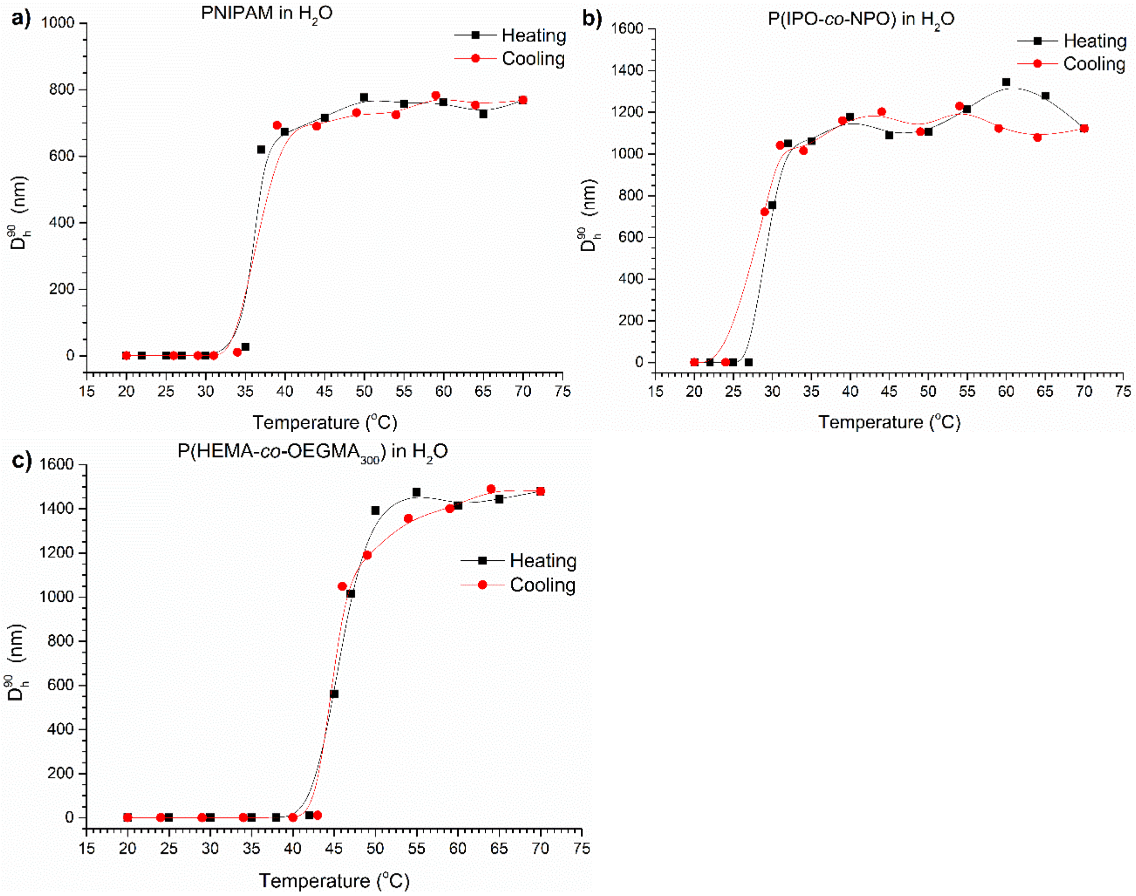

Figure S3. D_h^{90} versus temperature plots of water solution of a)PNIPAM, b) P(IPO-*co*-NPO), c) P(HEMA-*co*-OEGMA$_{300}$). Total solute concentration 0.5 g/L. Heating - black, cooling - red.

Table S1. Diameters of polymers aggregates after gradual heating depicted from DLS at 70 °C.

	Hydrodynamic diameter at 70 °C [nm]				
**Copolymer**	**Water**	**PBS**	**NaH_2PO_4 solution**	**Na_2HPO_4 solution**	**NaCl solution**
PNIPAM	770	4370	750	1540	4000
P(IPO-*co*-NPO)	1120	4000	1600	3100	3050
P(HEMA-*co*-OEGMA$_{300}$)	1480	3800	2230	3420	2650

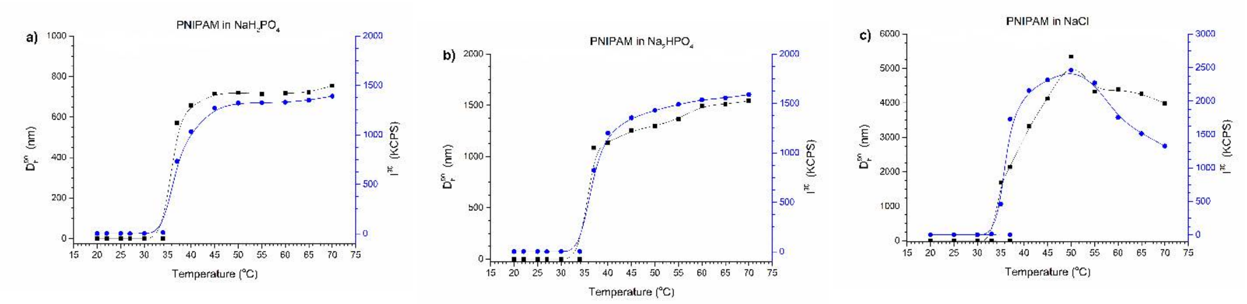

Figure S4. D_h^{90} and I^{90} versus temperature plots of PNIPAM in a) monosodium phosphate, b) disodium phosphate, c) sodium chloride solutions. Polymer concentration 0.5 g/L, gradual heating.

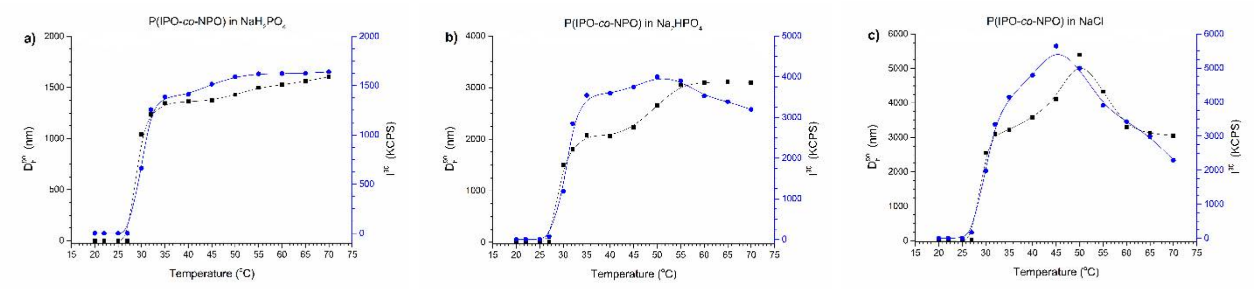

Figure S5. D_h^{90} and I^{90} versus plots of P(IPO-*co*-NPO) in a) monosodium phosphate, b) disodium phosphate, c) sodium chloride solutions. Polymer concentration 0.5 g/L, gradual heating.

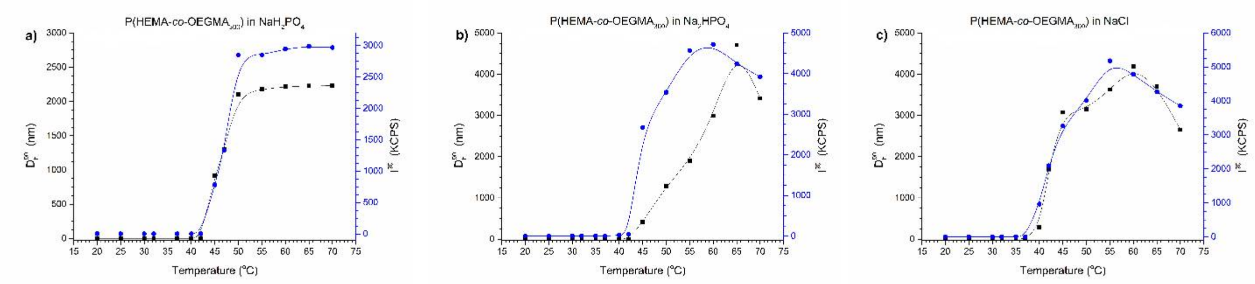

Figure S6. D_h^{90} and I^{90} versus temperature plots of P(HEMA-*co*-OEGMA$_{300}$) in a) monosodium phosphate, b) disodium phosphate, c) sodium chloride solutions. Polymer concentration 0.5 g/L, gradual heating.

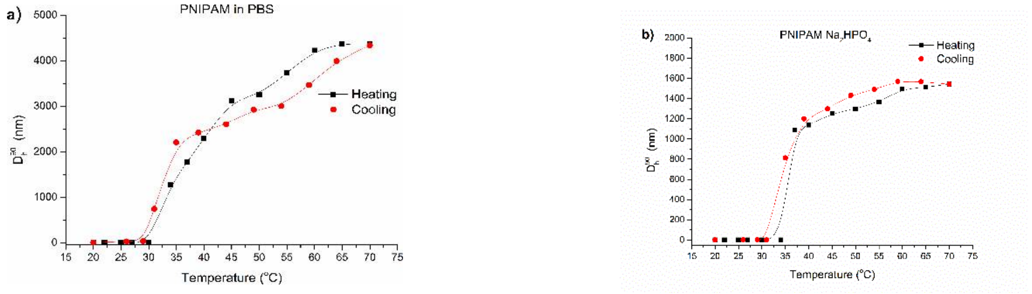

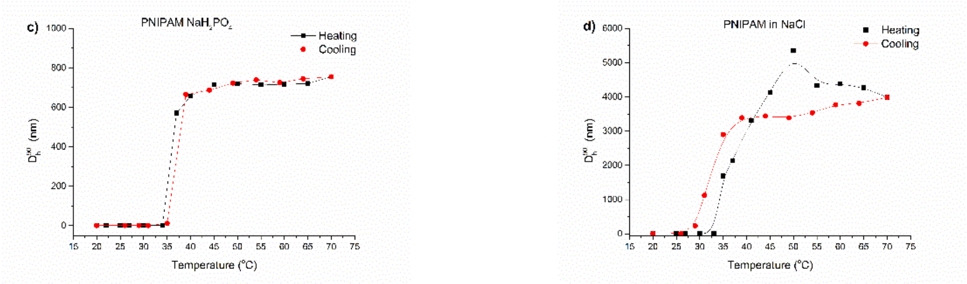

Figure S7. D_h^{90} versus temperature plots during heating and cooling of PNIPAM in a) commercial PBS b) disodium phosphate, c) sodium phosphate d) sodium chloride solutions. Polymer concentration 0.5 g/L, gradual heating.

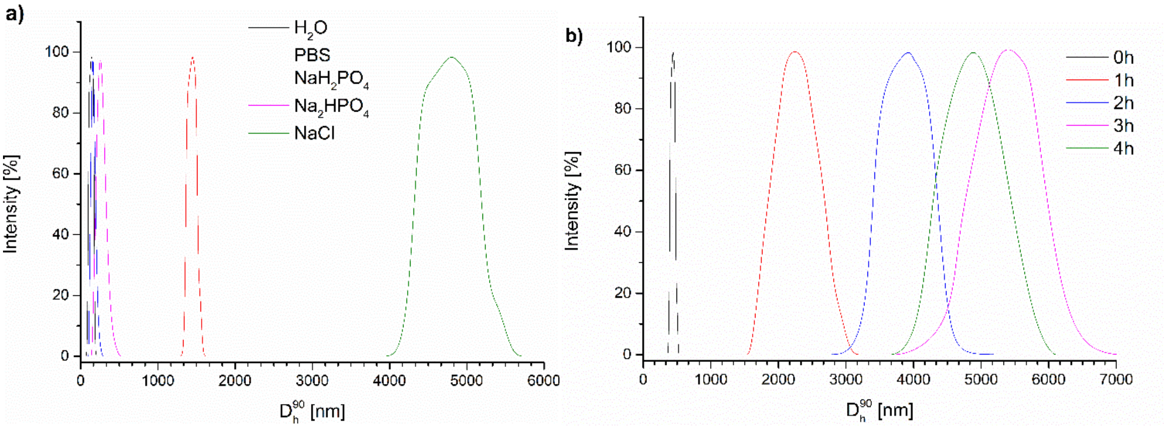

Figure S8. Particles size distribution of PNIPAM in abrupt heating a) in different solutions after 4h, b) in solution of NaCl after different times periods.
